# Is the destruction or removal of atmospheric methane a worthwhile option?

**DOI:** 10.1098/rsta.2021.0108

**Published:** 2022-01-24

**Authors:** Peter B. R. Nisbet-Jones, Julianne M. Fernandez, Rebecca E. Fisher, James L. France, David Lowry, David A. Waltham, Ceres A. Woolley Maisch, Euan G. Nisbet

**Affiliations:** ^1^ Department of Earth Sciences, Royal Holloway, University of London, Egham TW20 0EX, UK; ^2^ British Antarctic Survey, Natural Environment Research Council, Cambridge, UK

**Keywords:** methane removal, fugitive methane destruction, methane removal costs

## Abstract

Removing methane from the air is possible, but do the costs outweigh the benefits? This note explores the question of whether removing methane from the atmosphere is justifiable. Destruction of methane by oxidation to CO_2_ eliminates 97% of the warming impact on a 100-yr time scale. Methane can be oxidized by a variety of methods including thermal or ultraviolet photocatalysis and various processes of physical, chemical or biological oxidizers. Each removal method has energy costs (with the risk of causing embedded CO_2_ emission that cancel the global warming gain), but in specific circumstances, including settings where air with high methane is habitually present, removal may be competitive with direct efforts to cut fugitive methane leaks. In all cases however, great care must be taken to ensure that the destruction has a net positive impact on the total global warming, and that the resources required would not be better used for stopping the methane from being emitted.

This article is part of a discussion meeting issue ‘Rising methane: is warming feeding warming? (part 2)’.

## 1. Introduction: questions

Scenarios for mitigating climate warming driven by greenhouse gas emissions envisage steep and immediate reductions in atmospheric methane [1]. In the Summary for Policymakers, the Sixth Assessment Report of Working Group 1 of the Intergovernmental Panel on Climate Change advocated ‘Strong, rapid and sustained reductions in CH_4_ emissions' [2]. While cutting the methane burden is urgent and very beneficial [3,4], methane that has already been emitted can be removed from the air [5–7].

This paper uses some very simple estimates of the gain and costs of atmospheric methane removal in terms of global warming potential (GWP) (gCO_2_eq). These estimates involve simplified assumptions that necessarily affect their exactness, yet they are still considered valid in indicating the relative merits and potential viability of different approaches, as well as identifying important knowledge gaps.

The justification for methane removal is the high GWP of atmospheric methane. Over 100 years, removing 1 g of methane is equivalent, including feedbacks, to removing 34 g of CO_2_; however, over 20 years, 1 g of methane is equivalent to 86 g of CO_2_ [8]. If we are looking for a ‘quick win’—essential if we hope to keep heating below 1.5 degrees—methane is a very appealing target.

But what is the best option? Will generating the energy needed to remove the methane induce so much released CO_2_ that the net long-term greenhouse impact is warming, not mitigation? Or, given there will likely be a limit to the funding available to ameliorate greenhouse gas emissions, will it be better to use these resources to reduce methane emissions rather than to remove already-emitted methane from the air?

As the troposphere contains roughly 1900 parts per billion methane, there is a total burden of well over 5 × 10^3^ Tg (where a Teragram (Tg) is a million tonnes) of methane in the air. The molecular weight of air is nearly 29 g mol^−1^; sea-level pressure slightly over 1 bar; the radius of earth 6371 km. Thus, the mass of one part per billion (10^−9^) of methane in the air is about 2.77 Tg. The global methane budget is not balanced: the burden is growing because more is being added each year than is destroyed. In 2020, the growth of the methane concentration in the air was roughly 15 parts per billion [9], or over 40 Tg. In more human terms, this 40 Tg rise in 2020 is approximately as much methane as would be released if the global LNG tanker fleet was shipwrecked each year.

Estimates of methane sinks and sources vary. Based on atmospheric measurements (top-down), Saunois *et al*. [10] estimate total global annual methane emissions are 576 (range 550–594) Tg. Of this, anthropogenic methane emissions were estimated [10] at around 360 Tg, including well over 200 Tg emitted by agriculture, and well over 100 Tg emitted by the fossil fuel industry. However, isotopic evidence [11] suggests the fossil fuel industry may emit as much as 150–200 Tg methane annually. Nearly two-thirds of methane emissions are directly linked to human activities and are thus targets for mitigation.

Methane is naturally destroyed by both chemical and biological processes, including reaction with atmospheric hydroxyl [OH] and chlorine, and by methane-consuming bacteria (methanotrophs) in soil and water. This results in a lifetime in the air of 9.1 ± 0.9 years [12]. Thus, we face an important question—given methane is being removed from the air anyway, why trouble to do this artificially? It may be preferable to dedicate the cost and energy involved in methane removal to the task of stopping methane emissions, which would accomplish the same end result of lessening, halting, or reversing the growth of methane-driven climate warming, or, alternatively, simply to ignore methane and dedicate all efforts to CO_2_ removal. To answer this question, the specific methods of removing methane must be examined.

Methane removal from the air can be enhanced by a variety of techniques. But all methods of methane removal have energy and environmental costs and need capital investment. In order to achieve useful removal, it is necessary either to process a significant fraction of the atmosphere very cheaply, in less than a decade, or else carefully to prioritize targeting of specific air masses for methane removal in high concentration areas.

We must also distinguish between *destruction* and *extraction*. Due to methane's high GWP compared to carbon dioxide, in order to reduce most of the warming impact, it is not necessary to *extract* the gas (as is needed for CO_2_ removal) but simply to *destroy* it by oxidation. CO_2_ is produced by methane's oxidative destruction, but this has only roughly 3% of the 100-yr climate-warming potential. Put another way, methane destruction by oxidizing it to CO_2_ reduces its 20-yr warming impact by 99%, or, if considered on a 100-yr warming impact timescale, by 97%. Moreover, the waste CO_2_ may itself be later removed by atmospheric CO_2_ capture technologies under investigation elsewhere.

## 2. Targets for methane destruction

Atmospheric methane occurs in air masses at a wide range of concentrations. For simplicity, these should be considered in five categories, ranked by increasing concentration and rapidly decreasing prevalence.

1. Ambient air—around 1.9 ppm methane, available anywhere on the planet (about 0.1 ppm higher in the Northern Hemisphere than the Southern Hemisphere).
2. Methane-enriched air, around 10 ppm, for example in the wider vicinity of a group of cattle, directly above large area sources such as wetlands, rice paddies or landfill, or in the blast zone of an open-cast coal mine.
3. High-methane air, around 100 ppm, for example above a tank of manure or above feeding troughs in a barn holding a herd of dairy cattle.
4. Very high-methane air, 1000 ppm, for example near a gas-field dewatering installation, or a leaky compressor.
5. Near-explosive air, 1% methane, near a deliberately venting oil-field installation, leaking gas distribution or landfill gas extraction pipe, etc.

It takes energy to remove methane, and energy generation in most cases involves greenhouse gas emissions. If the use of energy in order to remove one warming unit (gCO_2_eq) of methane generates more than one warming unit of CO_2_, then there is no net gain. The numbers in table 1 thus present us with our targets. For methane *extraction*, any process that can remove a given concentration while emitting less than the *allowable cost*—the given equivalent mass of CO_2_ (gCO_2_eq)—will reduce the overall warming of the planet. For methane *oxidation*, as CO_2_ is emitted as a by-product, the allowable gCO_2_eq cost is reduced slightly, to 97–99% of that allowed for complete removal. As this is beyond the precision of any of the approximations presented here, the full gCO_2_eq number will be used for the following discussion.

**Table 1.** At given concentrations, the approximate mass of methane in one cubic metre of air at 0°C, and the mass of CO_2_ with an equivalent 20- or 100-yr global warming impact (using values from [8] Table 8.7 with climate-carbon feedbacks).

category	CH_4_ concentration (ppm)	mass of CH_4_ in 1 m^3^ of air	mass of CO_2_ with an equivalent 20-yr global warming impact (x86)	mass of CO_2_ with an equivalent 100-yr global warming impact (x34)
1	2	0.0014 g (1.4 mg)	0.12 g	0.05 g
2	10	0.007 g (7 mg)	0.6 g	0.2 g
3	100	0.07 g	6 g	2.4 g
4	1000 (0.1%)	0.7 g	60 g	24 g
5	10 000 (1%)	7 g	600 g	240 g

In addition to the simple energy balance one must consider if the methane removal provides the best marginal abatement cost. In any society, electricity will have various sources. If a new electricity demand is added in order to remove methane, then the electricity source that is relevant to the removal efficacy is the marginal source with the highest climate-warming emissions, not the lowest. Without the new energy demand for methane removal, the climate-warming output from the marginal electricity source would not have occurred. Correspondingly, even if the energy needed to destroy or remove methane is generated by an on-site non-CO_2_ electricity source such as a wind farm or solar array, that electricity might be better used to displace CO_2_ emission elsewhere, rather than for the task of methane mitigation. Thus, the proper comparator for evaluation of the relative benefit of methane destruction or removal is the *most* CO_2_ generating marginal energy source in the local power grid, *at that time*. In 2021, this comparator will be fossil fuel power stations, coal in particular, but in the future will hopefully be primarily wind, solar, hydro or nuclear power.

Consider a scenario in the early hours of a windy winter night in the United Kingdom, when there is excess electricity supply, which is 100% from wind and ongoing baseload nuclear generation. In this situation, and similar worldwide, there is often a negative marginal cost of electricity [13–15]. Destroying or removing methane then may be at very low or zero cost in gCO_2_eq. Now, second, consider an alternative extreme scenario on a cold anticyclonic afternoon when electricity demand is high and marginal coal-fired power is being imported from Europe. In this scenario, removing methane will increase coal-burning and will be very expensive indeed in gCO_2_eq. As energy storage methods improve these periods of the negative cost may be eliminated, but if intermittent methane sources—e.g. deliberate venting—could be processed during these periods, then processes with higher energy use would become acceptable. This could be a promising short-term avenue for research.

It is also necessary to consider the CO_2_ emissions of the infrastructure required to remove the methane, as it is the total CO_2_ emission that must be considered not just the running cost. For example, concrete manufacture is one of the major sources of anthropogenic CO_2_ emissions and so a removal technique which has high running costs but minimal infrastructure requirement may in practice be preferable to a highly efficient but infrastructure intensive removal process. This consideration is made particularly obvious in the case of methane destruction by solar UV radiation, which has no continuing energy cost, but the significant embedded gCO_2_eq from concrete in building a large-scale reactor, such as a solar updraft tower (SUT), must be accounted for.

Depending on the nature of that *most CO_2_ emitting* energy source in the local power grid, it is possible to estimate the allowable energy that can be used to remove methane from 1 m^3^ of air. A simple calculation of the beneficial running cost of any removal method is presented in table 2. For this analysis, the energy comparator for gas-fired electricity is derived from the 490 gCO_2_eq/kWh value for a natural gas combined cycle system, from Schlömer *et al*. [16]. Should the marginal power generator be coal-fired, then the value of 820 gCO_2_ eq/kWh is used as the appropriate comparator.

**Table 2.** Maximum allowable energy in kWh to remove methane from 1 m^3^ of air if net warming benefit is to be achieved. GWP equivalent values of gCO_2_ eq/kWh from Schlömer *et al*. [16] Table A.III.2, median values. A 20-yr timescale for CH4 : CO2 equivalence is chosen as displaying the strongest case for removal; if a 100-yr timescale is chosen, the case for removal is much weaker as the methane will have been oxidized by natural processes.

category	CH_4_ concentration (ppm)	wind (kWh) 11 gCO_2_eq/kWh	solar (kWh) 48 gCO_2_eq/kWh	gas (kWh) 490 gCO_2_eq/kWh	coal (kWh) 820 gCO_2_eq/kWh
1	2	0.01	0.003 (3 Wh)	0.0002 (0.2 Wh)	0.0001 (0.1 Wh)
2	10	0.05	0.01	0.001 (1 Wh)	0.0007 (0.7 Wh)
3	100	0.55	0.13	0.012	0.007 (7 Wh)
4	1000	5.5	1.3	0.12	0.07
5	10 000	55	13	1.2	0.7

The gCO_2_eq value for methane of the given concentration in 1 m^3^ of air is presented, along with the gCO_2_eq/kWh emission intensity for making electricity. To remove 10 000 ppm of methane from 1 m^3^ of air, it is net-beneficial if the removal process uses less than 50 kWh of wind-generated energy, but in contrast only the use of less than 0.7 kWh of coal-generated energy is net-beneficial. Removing methane from 1 m^3^ of ambient air (2 ppm) is beneficial only if this can be done with less than 10 Wh of wind power, or 0.1 Wh of coal power. Embedded infrastructure emissions from the power generation and reactor are neglected so the actual allowable energy limits will be lower than this. To put these numbers into context, 0.1 Wh will power an iPhone 12 for about 15 min. The values in table 2 thus suggest that air with 10 000 ppm methane is a potential candidate for mitigation by removal, while ambient 2 ppm air is not.

To address the practical options, there are many circumstances where habitually high-methane concentrations occur in hard-to-mitigate situations where emissions are intractable [17]. Obviously, it may be argued that it would be preferable to end the activities making the methane, but equally obviously there may be circumstances where that is technically or politically impossible. For example, close to cattle barns there are manure ponds and tanks, emitting Category 4 methane-rich air (approx. 1000 ppm), while inside the barns the breath of the cows may create Category 2/3 conditions (approx. 10–100 ppm). As it is hard to stop these emissions unless the whole operation is closed, ending the entire cattle farming industry, then such air may be a good target for mitigation by methane removal.

## 3. Potential methane removal methods

### (a) Whole atmosphere processing

Methane is relatively difficult to oxidize compared to other hydrocarbons. The major destruction options include (i) thermal-catalytic oxidation, which is typically with metal catalysts; (ii) photocatalytic oxidation; (iii) biological uptake by aerobic methanotrophic bacteria or their bio-engineered methane-oxidising enzymes and (iv) removal by uptake on zeolites or porous polymers, with the added benefit of not emitting CO_2_ waste.

In order to process the entire atmosphere within a specified time frame, for example one year, we can first estimate the scale of effort needed simply to move the air for this methane removal, not including the energy needed for the removal process itself. Consider a 3.5 m diameter fan driving air in a light breeze (2 ms^−1^) through the removal device. This would move nearly 20 m^3^ of air in a second, or approximately 10^9^ kg in a year. To move the entire atmosphere (5.2 × 10^18^ kg) in a single year would take 6 × 10^9^ such fans. If these were placed somewhere out of the way, but with excellent renewable energy supply—for example the Sahara desert which is about 10^7^ km^2^—that would need 400 solar-powered fans per km^2^, not exactly feasible, especially considering the cost of manufacture and upkeep, though arguably not outright impossible.

A less heroic and perhaps more realistic alternative to powered fans is to use SUTs [18,19]. The concept is that solar-heated air, produced beneath a transparent collector a few km across, escapes through a central chimney containing a wind turbine and provides low-tech, affordable renewable energy [20,21]. de_Richter *et al.* [22,23] investigated the use of SUTs for oxidation of non-CO_2_ greenhouse gases and demonstrated that plausible numbers of very large SUTs could generate electricity economically and also process a significant fraction of the atmosphere over a few decades. The suggested large SUT sizes are a consequence of optimizing for cheap power generation. Optimizing instead for atmospheric flux removes this requirement and allows much smaller and cheaper designs.

### (b) Thermal-catalytic oxidation

Perhaps the simplest method of methane destruction is catalyst-based oxidation by the passage of hot ambient air (approx. 400°C) over cheap catalysts like Hopcalite (Cu-Mn Oxides). This is the laboratory method commonly used to prepare ‘zero air’ (methane-free air) for research. The main energy cost in this process will be from heating ambient air to 400°C. Thermal energy can be recaptured by using exhaust gases to heat the intake, but nevertheless the energy cost is large. Air has a specific heat of about 1 kJ kg^−1^ K^−1^, so the heating needed to process the entire atmosphere, heating it to 400°C from an ambient 15°C, would require 1 × 385 × 5 × 10^18^ = 1.9 × 10^21^ kJ. Heat exchangers with near perfect efficiency exist, but even assuming an efficiency of 99% this would still correspond to an energy need of 2 × 10^19^ kJ.

The irony of heating the atmosphere in order to reduce global heating is not unappreciated. Even a 99% efficient heat exchanger would still result in 4°C global heating, which is far in excess of what we are trying to prevent.

This method may however be locally applicable if used where gas is already hot—for example around gas flares above oil wells, or just after a combustion chamber to remove waste gas in exhausts from gas-burning compressors, power generators or domestic gas boilers [24]. More sophisticated catalysts which may be able to operate at lower temperatures include noble metal catalysis (as used in car exhausts), orzeolite- or polymer-supported metal catalysts. Despite these possible advances thermal-catalytic destruction of methane in category 1–3 air is unlikely to be energetically viable, except in very unusual circumstances.

### (c) Photocatalytic oxidation

Photocatalytic oxidation of methane is similar to the natural atmospheric process that oxidizes CH_4_ into CO_2_. Ultraviolet light is used to split an oxygen molecule into two free radicals. These then react with the methane, to make products such as CH_3_OH, CH_3_OOH, HOCH_2_OOH, HCOOH, CO_2_ and water. In photocatalytic reactors, a catalyst such as zinc oxide [25] or titanium dioxide is used to increase the generation of free radicals, and thus the methane reaction rate. This is very attractive as ultraviolet light in sunlight can be used, for example on the roofs of cattle barns.

The problem is that photocatalysis is relatively slow. Zhu *et al.* [25] achieved rates of over 2 mmol g^−1^ per hour at atmospheric pressure and 50°C (easily achieved passively on a sunny day). We have not found published reaction rates for comparable photocatalysis of methane using common TiO_2_ catalysts. However, other hydrocarbons have been measured using TiO_2_ as the catalyst showing slower reaction rates for the smaller hydrocarbon molecules [26], and suggesting reaction rates at ambient, low concentrations of methane (around 1900 ppb), around 0.001 µmol c^−1^ m^2^-h at a UV-illumination energy of 0.9 mW cm^−12^ and an average water concentration of roughly 10 000 ppm. This is supported by some initial, unpublished, studies by the authors.

If all the rooftops on the planet were painted with white TiO_2_ paint, and the area of an average roof is 50 m^2^, then assuming 2 × 10^9^ buildings on Earth gives a surface area of 1 × 10^11^ m^2^. Assuming bright sunlight for 6 h per day, over 100 days a year, at an oxidation rate of 0.01 moles m^−2^ h^−1^, a rough calculation suggests nearly 10 Tg of methane could be destroyed annually; for comparison the year-on-year methane increase in 2020 was roughly 40 Tg. It should be noted that white roofs have both positive and negative impacts, with the albedo benefit of reflecting sunlight back to space, but also the reflected light affecting urban pollutants [27]. Roofs are very widely painted so, compared to present-day emissions, the incremental CO_2_ emission in mining TiO_2_ and manufacturing paint could be small.

The development of active photocatalytic reactors is limited not by the energy cost—high-quantum efficiency LEDs can generate the required illumination—but instead by the low generation rate of OH radicals per unit area of TiO_2_. Advances in materials science improving inexpensive oxide semiconductor photocatalysts [25] could have a very large impact here, with the potential to enable high flow-rate reactors to process air masses in all methane concentration categories. Thus, photocatalytic oxidation is a strong potential target for research.

### (d) Biological oxidation

Biological methanotrophic methane removal from the air occurs widely in nature, where methanotrophs are ubiquitous in the global biosphere. In specific settings, for example in well-aerated moist forest soils, methanotrophic removal plays a major role in microbial consortia. In settings where methane concentrations are higher, as in termite mounds, aerobic methanotrophs can remove half the methane emissions [28]. Biofiltration of high concentration methane (% level) is a well-established technique [29]. Thus, discovery and utilization of the specific metabolic capabilities of methanotrophic communities offer much potential in developing bacterial methane removal technology.

Soil methanotrophs are capable of removing ambient methane (greater than 2 ppm) [30] when natural meteorological fluctuations in air pressure pump air slowly through moist aerated soils, for example in upland forests. These natural processes make soil methanotrophy a significant global methane sink [10]. However, uptake rates per m^2^ are very slow. Presumably uptake could be enhanced, for example by watering desert soils, but this would take large amounts of energy, destroy ecosystems, and only slightly increase the uptake of methane by the world's soils. It is thus unlikely there would be net global warming benefit.

Methane egresses over managed landfills from active faces, from breaks and leaks in gas piping, and through cover soils. Estimates are that greater than 10% of methane that migrates to the top of a landfill is oxidized in the cover soils in well-managed landfill sites [31]. Currently, many tropical urban landfills are little managed, and egress fluxes unknown but potentially large. In these neglected landfills, adding more cover soils should be a simple but effective passive management technique of methane removal, providing host soil for methanotrophic uptake and also reducing smell and the risk of fires, which create local urban pollution.

Study of methanotrophy at the vents of termite mounds has shown that specific highly adapted communities of methanotrophic bacteria may substantially consume the low concentrations of methane emitted by the termite nests (Category 2–3), with specialized bacteria [32] oxidizing perhaps 50 ± 11% of the methane [28]. This surprising finding suggests research into methanotrophic communities responsible for methane uptake in termite nests may provide a useful biological option for methane removal in settings such as landfills and around cattle barns, where local air masses are likely comparable to termite nests in methane concentration. In general, there is much scope for research into effective strains of methanotrophs capable of efficient methane removal from lower concentration air, for example, using methanotrophic biotrickling filters [33].

There is, however, a serious concern that has received little attention—the incidental manufacturing of other greenhouse gasses. If nitrogen compounds are present in the ‘dirty’ air, for example, high ammonia concentrations in emissions from animal waste, the biofilter can oxidize them to N_2_O, a gas with a long lifetime and GWP 300 times that of CO_2_. Bio-reactors can emit significant volumes of N_2_O [34,35]. Melse *et al*. [35] showed that in one case over 20% of all ammonia present was converted to N_2_O and thus cautioned against recommending biofilter installations at livestock operations. This problem must be solved before biological methane destruction can be viable.

### (e) Zeolite or membrane extraction methods

Oxygen concentrators are widely used in medicine and have been important in the COVID-19 pandemic. Oxygen concentrators use molecular sieves to adsorb nitrogen onto zeolites, scrubbing the nitrogen from the air and thus concentrating the remaining oxygen for the patient. Rapid pressure swing adsorption [36] is used, with the nitrogen being adsorbed at high pressure, so that the exiting gas is rich in oxygen. The nitrogen is desorbed at low pressure and vented, before the start of the next adsorption cycle. With two linked systems, a steady oxygen flow can be maintained to the patient.

Comparable systems might be used to concentrate and extract methane. For example, zeolite-based extraction of methane is feasible [5,37], with the added possibility that with embedded metal ions in the zeolite it may be possible to oxidize the sorbed methane and thus release it as CO_2_. Methane extraction by gas separation membranes [38] is another potentially feasible method of methane removal. These methods rely on techniques that are already in widespread industrial use that could likely be readily upscaled in a way that a more experimental technology could not, which is a significant advantage when dealing with the immediate need for decarbonization. However, despite this promise, there is a pressing need for analysis into their net energy balance and gCO_2_eq outcomes.

## 4. Examples of locations where methane removal is feasible

### (a) Landfills

Methane removal and destruction by combustion from landfill gases is well established and has greatly contributed to cutting European methane emissions. This is done physically, by collecting the gas and piping it to combustion for electricity or to be sold as ‘green gas’. But there is wide scope for more attention to locations in landfills where methane is present but not at concentrations that are combustible, in particular biological mitigation by soil bacteria. The addition of soil covers that have been seeded with highly efficient methanotrophs is also an extremely promising approach in regions where the capital outlay for piped gas collection and combustion is not affordable, for example in landfills around rapidly growing tropical cities.

Moreover, methane is not usually collected from the active uncovered surfaces of landfill cells, where new waste is being added. In such locations, it may be feasible to design low-cost removal techniques such as zeolite-based extraction to mitigate high-methane air around the active face of the landfill.

### (b) Biodigesters

Anaerobic biodigesters are increasingly common across Europe, where domestic waste is widely sorted for organic matter such as food waste. This markedly reduces landfill size and emissions, but biogas facilities often have significant methane emissions, which are only lightly regulated. Bakkaloglu *et al*. [39] estimate, with wide error bounds, that perhaps nearly 2% of UK methane emissions come from biogas plants. Part of this emission will come from accidental leakage, but normal management practice should be improved to include better leak detection, with likely wide circumstances where methane removal is advantageous from high-methane ambient air close to anaerobic tanks.

### (c) Fossil fuel industry facilities

The natural gas industry has widespread locations where the air is habitually present with Category 3–4 air, i.e. high but not combustible methane concentrations [17]. For example, this is likely around dewatering systems, near gas-gathering or compressing installations, and in urban reticulation around gas governors. In all these settings, methane removal is likely feasible. Oil wells frequently flare unwanted gas, with the emission of unburnt methane [40,41]. Here regulatory changes to operating procedures could enforce photo- or thermal-catalytic destruction of methane that escapes the flame.

Likewise regulations about catalytic converters for NOx on methane combustors, be they gas turbines or domestic boilers, could be updated to include the oxidation of fugitive methane in the exhaust.

### (d) Cattle

Dairy cattle in the United Kingdom typically rotate between open fields and cattle barns. In the cattle barns, category 2/3 air (10–100 ppm) close to feeding troughs is a potential target for methane removal efforts. Manure from grass-fed cattle on open fields is impossible to capture but soon dried, but in cattle barns, manure is collected as a slurry in anaerobic pipes and tanks. One option is deliberately to use the manure in sealed biodigesters to make methane, and then burn it to generate electricity. Where this is not feasible, high-methane air around manure slurry facilities is a major target for methane removal. Biological treatment methods are already common in the pig farming industry [42] and can be adapted to methane removal if the issues around N_2_O generation can be resolved. Photocatalytic oxidation is possible, however, the high concentration of other organic molecules which will be preferentially oxidized before methane will limit its effectiveness. Similarly, beef feedlots are major methane sources [17] and removal could mitigate manure emissions, though a better long-term option might be to rear only free-range grass-fed beef where the manure is rapidly dried in open pasture.

## 5. Removal or emission reduction?

To summarize: removal is feasible, but probably only justified in special circumstances where high-methane concentrations are habitually present. Removal by thermal oxidation is likely only justifiable where methane-rich hot gas is already present, for example in hot exhausts of gas-fired compressors or heating systems. Removal by photocatalysis may be feasible, but without the development of high-surface area catalysts is perhaps useful in the near future only in cases such as sunlight on photocatalytic paint where surfaces such as roofs are already intended to be painted. By contrast, passive biological removal, for example by landfill cover soils, may be very widely justified, especially in the Tropics and even more so if more effective strains of methanotrophs can be found.

Alternatively, a radical proposal is for removing methane by blowing iron-salt aerosols into the troposphere [43] in order to enhance the Cl sink. But as with all geo-engineering, there are ethical objections: is it right to pollute the global commons, the air, to alleviate the need to reduce pollution that is emitted by specific groups? The many scientific uncertainties around ##geo-engineering contribute to the ethical debate however the political issues are also often overlooked. Principle 2 of the Rio Declaration [44] imposes ‘the responsibility to ensure that activities within their jurisdiction or control do not cause damage to the environment of other States or of areas'—an international law that geo-engineering would contravene. The ethics of adding cross-border geo-engineering pollutants to mitigate cross-border global warming pollutants may become a major issue in the coming decades. Intuitively, it is better not to have a problem than to deal with it. Stopping the emission of methane is preferable to removing it from the atmosphere. Many methane emissions, such as deliberate venting or dewatering from oil and gas industry installations, venting from biodigesters and emissions from uncapped landfills, are easily prevented by better design and regulation [17]. Methane leaks from gas pipes are easily located with modern instruments [45]. Once located these are relatively cheaply fixed, and pipeline replacement by threading a new pipe inside the old is a well-understood technology [17].

The problem is capital cost, and in some cases regulatory framework, as regulators can hinder pipeline renewal where it increases the capital cost of the regulated industry and hence the cost to the consumer [46]. Thus, at times there are contradictory pressures—one arm of government struggling to reduce emissions, while another arm of government encourages activities that promote them. Methane removal may increase N_2_O release. It is also feasible that some tax initiatives could favour complete methane removal, while simultaneously other policies block much less expensive leak reduction.

Given the complexities, it will be hard to find an optimum path towards the reduction of any country's net total national methane output. Thus, the arguments presented in this paper can be condensed into ‘Three Laws of Removal’. As with Asimov's Laws of Robotics, these are simple guiding principles to which any process should adhere, even if their exact implementation is not so clear-cut :

1. *Removal must do no worse harm*—i.e. the removal process must not cause worse effects than simply leaving the methane be, for example, the emission of a long-lived pollutant such as N_2_O, toxic organic compounds, or the permanent and irreparable damage to global ecosystems against which we have no mitigation.
2. *Removal must reduce net global warming*—i.e. the total gCO_2_eq captured must be more than that created to accomplish the removal—including all embedded and power generation emissions.
3. *Removal must be more cost-effective than reduction*, at least in a local setting. When limited resources are available they must be used in the most effective way, even if this is routine, low publicity, work such as replacing broken pipes. Evaluation must also include a comparison of the social and economic costs between removal and simply stopping the emitting activity completely.

It is clear that emission reduction and removal both need much research, both in wider social terms—should domestic gas distribution be replaced by electric heating, even if methane capture devices can be fitted to remove emissions from boiler exhausts and distribution networks?—and on very specific scientific problems—how best to remove methane from specific target air mixtures? There are no clear answers, but we hope the simple estimates presented here can help guide investigation into the most helpful choices.

## Data accessibility

This article does not contain any additional data.
