# Correction to: ‘Is the destruction or removal of atmospheric methane a worthwhile option?’ (2021) by Nisbet-Jones *et al.*

**DOI:** 10.1098/rsta.2023.0258

**Published:** 2023-11-27

**Authors:** Peter B. R. Nisbet-Jones, Julianne M. Fernandez, Rebecca E. Fisher, James L. France, David Lowry, David A. Waltham, Ceres A. Woolley Maisch, Euan G. Nisbet

**Keywords:** methane removal, fugitive methane destruction, methane removal costs

*Phil. Trans. R. Soc. A* **380**, 20210108. (Published online 6 December 2021). (doi:10.1098/rsta.2021.0108)

In the original version of this paper, a typographical error meant that the mass of the methane burden was incorrectly stated as 5 × 10^9^ Tg instead of 5 × 10^3^ Tg. This has now been corrected.

